# CLIC1 and CLIC4 complement CA125 as a diagnostic biomarker panel for all subtypes of epithelial ovarian cancer

**DOI:** 10.1038/s41598-018-32885-2

**Published:** 2018-10-03

**Authors:** Bipradeb Singha, Sandra L. Harper, Aaron R. Goldman, Benjamin G. Bitler, Katherine M. Aird, Mark E. Borowsky, Mark G. Cadungog, Qin Liu, Rugang Zhang, Stephanie Jean, Ronny Drapkin, David W. Speicher

**Affiliations:** 1 Molecular and Cellular Oncogenesis Program, The Wistar Institute, Philadelphia, Pennsylvania 19104 USA; 2 Department of Obstetrics and Gynecology, University of Colorado, Aurora, Colorado 80045 USA; 3 Department of Cellular and Molecular Physiology, Penn State College of Medicine, Hershey, Pennsylvania 17033 USA; 4 Helen F. Graham Cancer Center & Research Institute, Newark, Delaware 19713 USA; 5 Gene Expression and Regulation Program, The Wistar Institute, Philadelphia, Pennsylvania 19104 USA; 6 Department of Obstetrics and Gynecology, Ovarian Cancer Research Center, University of Pennsylvania Perelman School of Medicine, Philadelphia, Pennsylvania 19104 USA

## Abstract

New plasma and tissue biomarkers of epithelial ovarian cancer (EOC) could improve early diagnosis and post-diagnosis clinical management. Here we investigated tissue staining and tissue secretion of CLIC1 and CLIC4 across EOC subtypes. CLIC1 and CLIC4 are two promising biomarkers we previously showed were elevated in EOC patient sera. Individually, CLIC1 or CLIC4 stained larger percentages of malignant tumors across all EOC subtypes compared with CA125, particularly early stage and mucinous tumors. CLIC4 also stained benign tumors but staining was limited to nuclei; whereas malignant tumors showed diffuse cellular staining of stromal and tumor cells. Both proteins were shed by all EOC subtypes tumors in short term organ culture at more consistent levels than CA125, supporting their potential as pan-subtype serum and tissue biomarkers. Elevated CLIC4 expression, but not CLIC1 expression, was a negative indicator of patient survival, and CLIC4 knockdown in cultured cells decreased cell proliferation and migration indicating a potential role in tumor progression. These results suggest CLIC1 and CLIC4 are promising serum and tissue biomarkers as well as potential therapeutic targets for all EOC subtypes. This justifies development of high throughput serum/plasma biomarker assays to evaluate utility of a biomarker panel consisting of CLIC1, CLIC4 and CA125.

## Introduction

Epithelial ovarian cancer (EOC) consists of four major subtypes (serous, clear cell, endometrioid, and mucinous) that are quite different from each other at the genetic level, and most previously identified biomarkers differ significantly between subtypes^1^. When EOC is diagnosed early, 5-year survival of patients is close to 90%. However, most cases are not detected until after the cancer has metastasized, resulting in a dismal overall 5-year survival rate of less than 30%, corresponding to about 16,000 deaths annually in the US alone^2–4^. Current screening methods for EOC include pelvic examination, transvaginal ultrasonography and evaluation of serum levels of cancer antigen 125 (CA125)^5–7^. CA125, also known as MUC16, is the most commonly used EOC serum/plasma biomarker. It was discovered more than three decades ago^8^ and is an FDA approved EOC biomarker. Unfortunately, CA125 is inadequate as an early screening biomarker for at risk populations due to low sensitivity and specificity. The poor sensitivity of CA125 is primarily due to the fact that most early stage and low-grade tumors as well as mucinous subtype tumors and approximately 20% of advanced EOC cases do not show significantly elevated CA125 levels^9–13^. Furthermore, CA125 is not highly specific, as it is elevated in some benign EOC tumors and in non-cancerous conditions such as diverticulitis, liver cirrhosis, menstruation, uterine fibroids, and pregnancy^14^. There are also several other FDA approved EOC biomarkers that have specific uses, including: human epididymis protein-4 (HE4), which is primarily used to monitor therapeutic responses and disease reoccurrence, and multivariate OVA1, which is used to recommend the most appropriate type of surgeon for women diagnosed with an ovarian adnexal mass^15,16^. A number of potentially promising biomarkers were recently described^17–22^ but none outperformed CA125^23,24^. Thus, there is an urgent need to identify better biomarkers that will improve EOC diagnosis, either as a replacement for, or more likely, as a complement to CA125.

Our long-term goal is to identify biomarkers that can improve diagnosis of EOC across all major subtypes, particularly biomarkers that may be useful for both plasma/serum and tissue imaging. We previously showed, using a mouse xenograft model, that two members of the chloride intracellular channel (CLIC) protein family, CLIC1 and CLIC4, were produced in xenografted mice by implanted human ovarian tumor cells and shed into the blood^25,26^. Levels of both proteins were significantly elevated in sera from ovarian cancer patients compared with normal and benign controls, with receiver operating characteristic curves having an area under the curve of 0.79 for CLIC1 and 0.86 for CLIC4^25,27^. A more recent, independent report also described CLIC1 as a biomarker for detecting intraperitoneal metastasis of serous EOC^28^. Other studies have indicated that CLIC1 and CLIC4 may play roles in altering the EOC tumor microenvironment and affecting EOC tumor progression. CLIC4 regulated TGFβ-mediated activation of fibroblasts in EOC^29^ and knocking down CLIC1 arrested EOC cell division at the G1 phase, inhibited xenograft growth *in vivo*, and increased susceptibility of the EOC cells to cisplatin and ROS exposure^30^.

CLIC1 and CLIC4 have also been implicated in other cancers. In pancreatic cancer^31,32^ and glioblastoma^33,34^, CLIC1 overexpression was associated with poor prognosis and its inhibition reduced pancreatic cancer cell proliferation^32^. Similarly, treating glioblastoma cells with CLIC1 containing extracellular vesicles increased glioblastoma cell proliferation both *in vitro* and *in vivo*^34^. CLIC1 expression in gall bladder^35^ and hepatic cancers^36^ correlated with tumor progression and poor disease prognosis. In colorectal^37^ and prostate cancer^38^, CLIC1 inhibition decreased cell migration and proliferation followed by a subsequent reduction in the levels of p-ERK, MMP2 and MMP9 suggesting possible roles of CLIC1 in inducing tumor progression through MAPK/ERK and MMP mediated pathways. CLIC4 expression has also been shown to correlate with cancer metastasis and poor clinical outcomes^39,40^ and inhibiting CLIC4 expression decreased proliferation of glioblastoma^41^, head and neck cancer^42^ and osteosarcoma^43^ cells. In some cancers such as cutaneous squamous cell cancer^44^ and lung cancer^45^, however, induced CLIC4 expression decreased proliferation of these tumor cell types, suggesting CLIC4 may have tumor suppression roles in some situations.

In this study we evaluated the utility of CLIC1 and CLIC4 as EOC tissue biomarkers because we previously showed they are promising EOC serum biomarkers^25,27^ and have intriguing, but incompletely understood roles in EOC tumor progression. This study was designed to complement our prior human serum biomarker studies in part because it is very difficult to quantitate low abundance biomarkers in blood of large numbers of patients unless robust ELISA assays are available. Specifically, IHC can provide additional insight as to whether proteins of interest are produced by all EOC subtypes in parallel with determining their potential utility as tissue biomarkers. Both CLICs stained tumor tissues from all EOC subtypes, including early stage and low-grade tumors, which resulted in better sensitivity than CA125 alone at the tissue level. Also, an exploratory tissue secretome analysis showed that CLIC1 and CLIC4 were shed in short term organ culture by all EOC subtypes and levels of shedding were usually more consistent than CA125 shedding. Furthermore, consistent with other reports, these proteins appeared to play important roles in EOC progression across multiple EOC subtypes. Elevated CLIC4 expression was a negative indicator of patient survival, and knockdown of either CLIC4 or CLIC1 in cultured cells significantly decreased cell migration and colony formation. These results suggest that a biomarker panel consisting of CLIC1, CLIC4 and CA125 should outperform individual biomarkers for staining ovarian tumor specimens and could potentially be used in a serum screen assay for all EOC subtypes. Taken together with the prior patient serum studies, the IHC and tumor secreteome strongly indicate that investing the needed effort and cost to develop high throughput serum/plasma assays for CLIC1 and CLIC4 are justified.

## Results

### Specificity of CLIC1 and CLIC4 as EOC biomarkers

**Figure 1:**
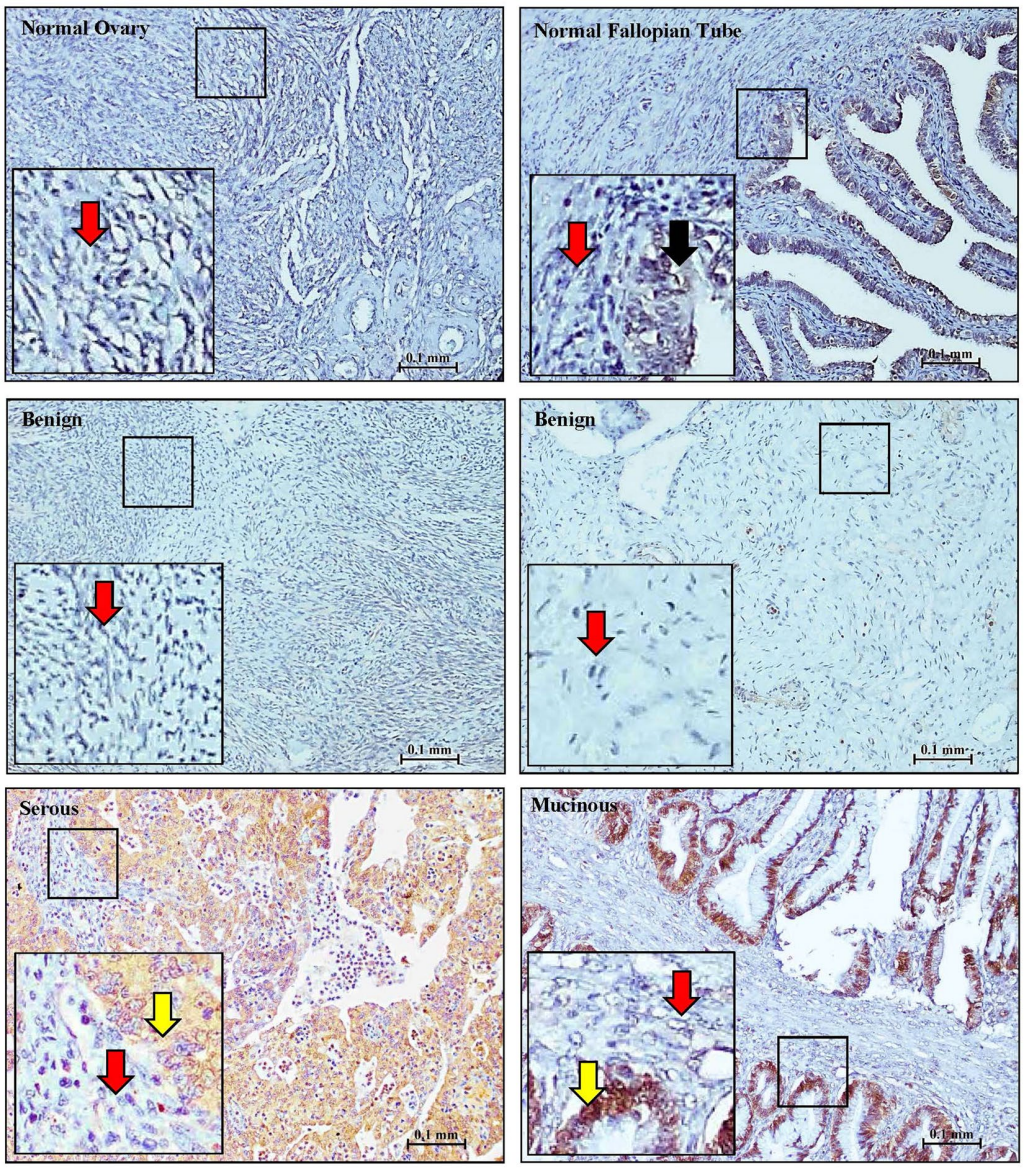
CLIC1 staining in normal, benign and EOC tissues. Representative images of CLIC1 staining of normal ovary, fallopian tubes, benign tumors, and serous (stage IV) and mucinous (stage I) EOC tissue. Red arrows = stroma, black arrows = normal epithelial cells and yellow arrow = tumor cells. Locations of high magnification insert are indicated by the black box.

**Figure 2:**
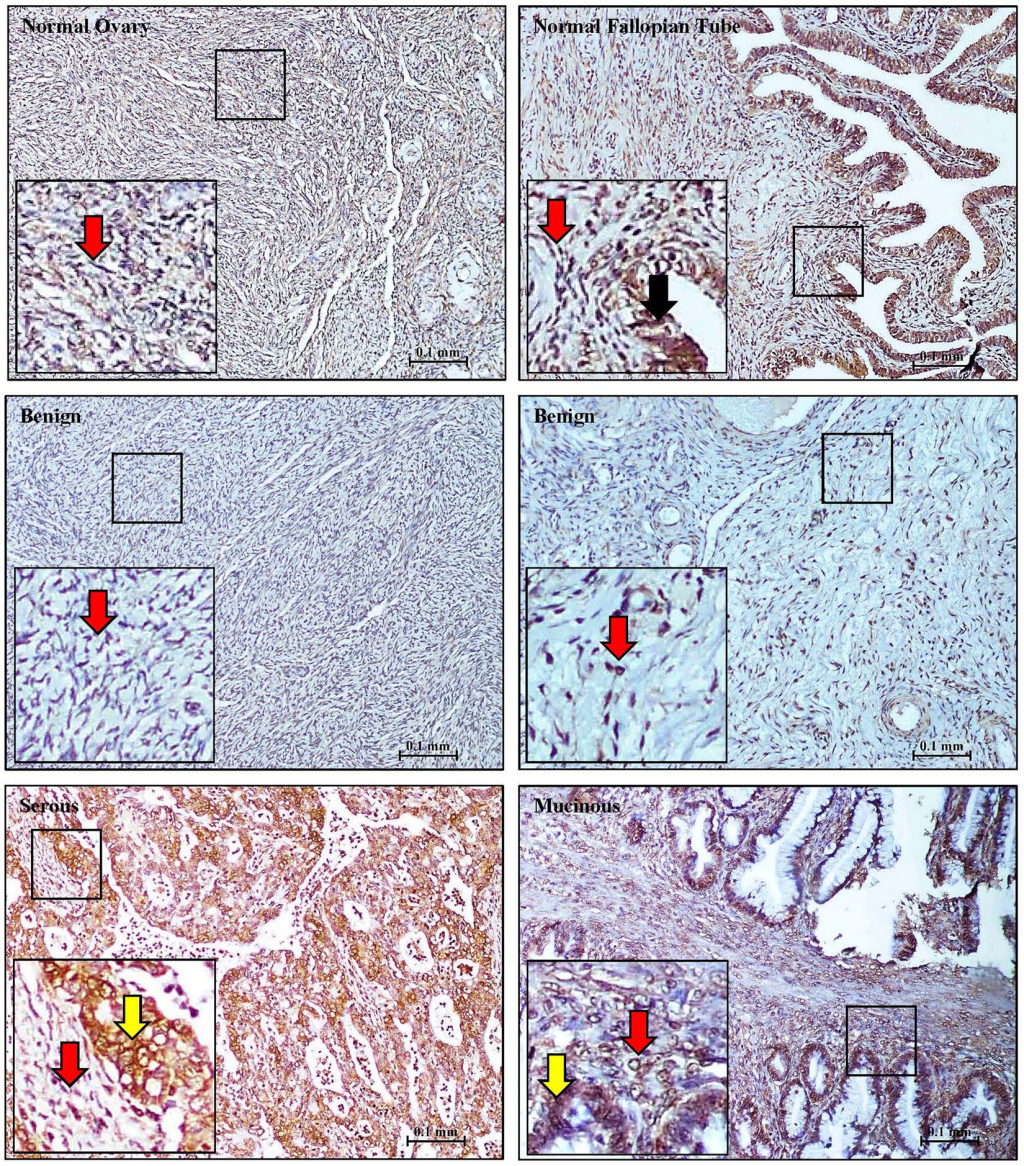
CLIC4 staining in normal, benign and EOC tissues. Representative images of CLIC4 staining of normal ovary, fallopian tubes, benign tumors, and serous and mucinous EOC tissue. Tissue cores are identical to the ones used in Fig. 1. Red arrows = stroma, black arrows = normal epithelial cells and yellow arrow = tumor cells. Locations of high magnification insert are indicated by the black box.

**Figure 3:**
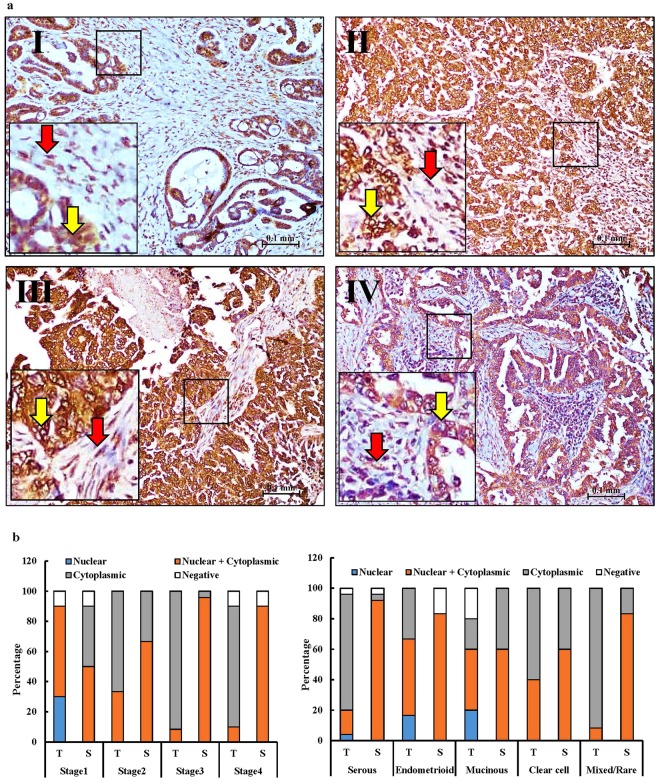
Changes in CLIC4 staining at different EOC stages. (**a**) Representative images of CLIC4 staining in stage I, II, III and IV serous EOC tumors. Red arrows = staining in the stroma and yellow arrow = staining in tumor cells. (**b**) Percentage of tumor nests (T) and stroma (S) that show the indicated distribution of nuclear, cytoplasmic, nuclear + cytoplasmic or negative staining for CLIC4 across different EOC stages and subtypes.

**Figure 4:**
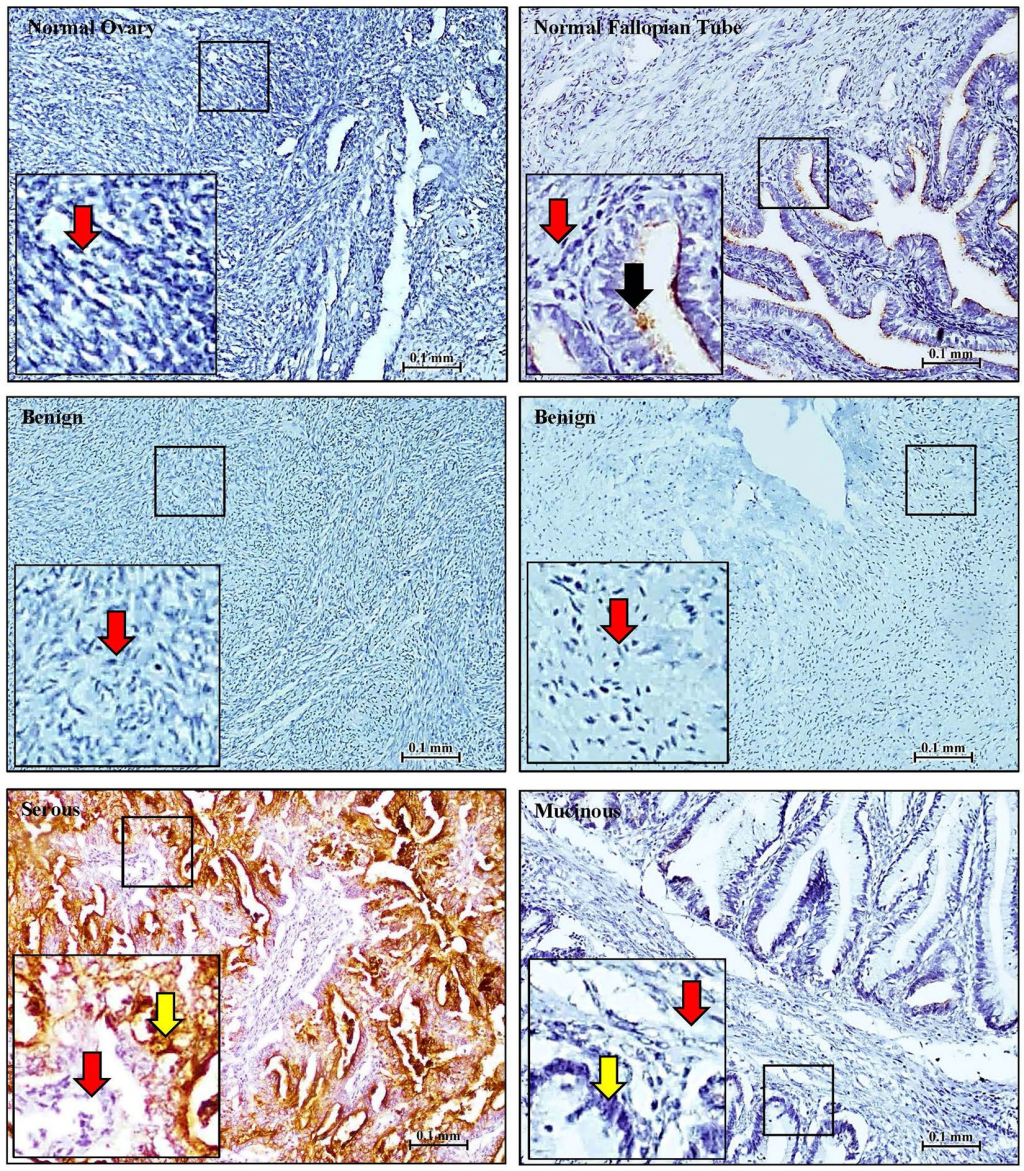
CA125 staining in normal tissues, benign tissues and EOC tissues. Representative images of CA125 staining in the normal ovary, fallopian tubes, benign tumors, and serous and mucinous EOC tissue. Tissue cores are identical to the ones used in Fig. 1. Red arrows = stroma, black arrows = normal epithelial cells and yellow arrow = tumor cells. Locations of high magnification insert are indicated by the black box.

To evaluate the levels and distribution of CLIC1 and CLIC4 in ovarian tissues, we performed immunohistochemistry (IHC) staining on tissue microarrays containing normal (5 normal ovary and 4 normal fallopian tube), benign ovarian tumors (n = 11) and malignant ovarian tissues (n = 55) using monoclonal antibodies to CLIC1, CLIC4 and CA125. Staining was quantified using intensity alone rather than considering both intensity and stain distribution because, in general, staining intensity was relatively uniform across entire tissue cores (Supplementary Fig. S1). As shown in Fig. 1, normal ovaries and benign tumors did not typically stain for CLIC1. Normal fallopian tubes had only very weak apical CLIC1 staining within the epithelial lining and negative staining of stromal cells. In contrast, the tumor nests in most EOC tumors were positive for CLIC1, while stromal cells were negative. The same tissue specimens exhibited a more complex staining pattern for CLIC4. Normal ovaries, fallopian tubes, and benign tumors showed uniform, light CLIC4 staining that was primarily nuclear. In contrast, most malignant tumors showed strong CLIC4 staining in both tumor nests and adjacent stroma (Figs 2 and 3). Interestingly, CLIC4 staining patterns varied based upon cancer stage and tumor subtype (Fig. 3). In contrast to normal and benign tissues, no malignant tumors showed exclusively nuclear staining. While a few stage 1 tumors showed only nuclear staining in tumor nests, they also showed either cytoplasmic or both cytoplasmic and nuclear staining of the stroma (Fig. 3B). As the tumor stage increased, there was a steady shift toward primarily cytoplasmic staining in tumor nests, whereas stroma typically showed strong staining of both nuclei and cytoplasm. Moderate differences in staining patterns across cancer subtypes were similarly observed with staining in tumor nests of serous and mixed/rare subtypes typically limited to cytoplasmic staining, whereas most stroma showed strong staining in both nuclei and cytoplasms. Analysis of CA125 staining showed that, like CLIC1, CA125 was usually not detected in normal ovaries or benign tumors, whereas epithelial cells of the fallopian tubes stained weakly. Most EOC subtypes stained strongly positive for CA125 with the exception of mucinous tumors, which were always negative, consistent with previous reports^12,13^ (Fig. 4).

Meta-analysis of gene expression levels showed that the mRNA levels of CLIC1 and CLIC4 in normal ovarian surface epithelium, fallopian tube epithelium and ovarian tumors were consistent with the IHC staining patterns (Supplementary Fig. S2). Specifically, CLIC1 mRNA levels were very low in normal ovarian surface epithelium and fallopian tube epithelium, while levels in high grade serous ovarian tumors were significantly higher. Similarly, CLIC4 expression levels were significantly higher in high grade serous tumors compared with normal ovarian surface epithelium or fallopian tube epithelium. As expected, mRNA levels of CA125 were low in the normal tissues and significantly higher overall in the high grade serous ovarian tumors (Supplementary Fig. S2c).

### Sensitivity of CLIC1 and CLIC4 as EOC tissue biomarkers

**Figure 5:**
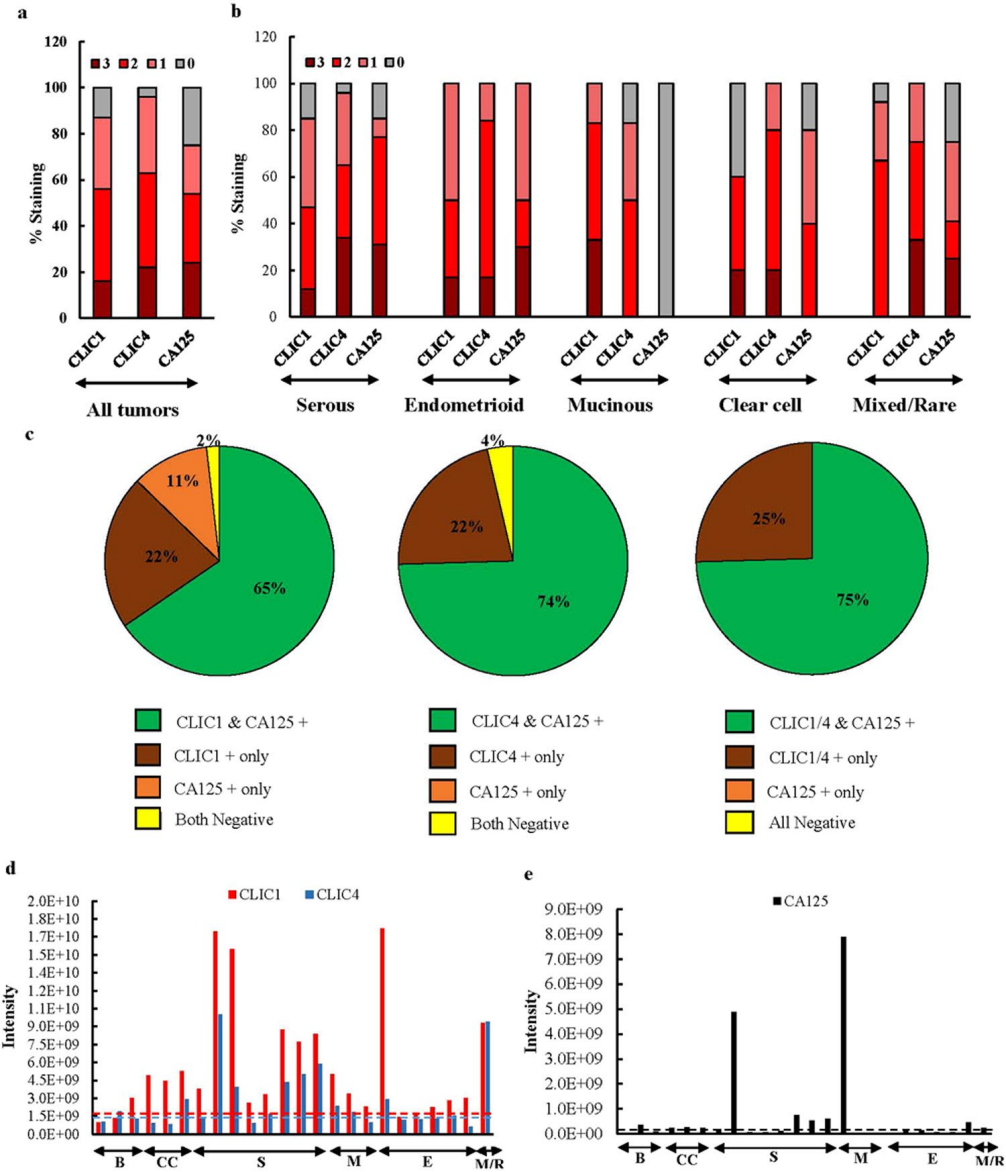
Sensitivity of CLIC1 and CLIC4 as EOC biomarkers. Intensity of CLIC1, CLIC4 and CA125 staining across all tumors (**a**) and tumors of each EOC subtype (**b**). (**c**) Complementarity among CLIC1, CLIC4, and CA125 staining. (**d**) Quantitation of CLIC1 and CLIC4 shed from benign and malignant ovarian tumors expressed as protein intensity; B = Benign (n = 3), CC = Clear cell (n = 3), S = Serous (n = 8), M = mucinous (n = 3), E = Endometrioid (n = 6), M/R = Mixed/Rare (n = 1). The red (1.8E + 9) and blue (1.4E + 9) horizontal lines indicate the average secretion of CLIC1 and CLIC4, respectively, from the benign tumors. (**e**) Quantitation of CA125 from the same secretomes as in panel d. Black horizontal line (1.5E + 8) indicates the average CA125 shed from the benign tumors.

To evaluate the sensitivity of CLIC1 and CLIC4 staining for malignant tumor cores, we categorized the tumors based on their staining intensities and compared tumor subtypes. Across all malignant tumors, CLIC1 and CLIC4 stained 87% and 96% of the tumor cores, respectively, while CA125 stained only 75% (Fig. 5a). When these data were organized by EOC subtypes, the most striking difference between the CLICs and CA125 was observed for mucinous EOC tumors, which were uniformly positive for CLIC1, 83% positive for CLIC4, and uniformly negative for CA125. Although less striking than the mucinous subtype, CA125 also stained “mixed/rare” tumors less consistently than the other two biomarkers, while CLIC1 stained clear cell tumors less consistently than the other two biomarkers and endometrioid ovarian tumors were uniformly positive for all three biomarkers (Fig. 5b).

We next evaluated whether CLIC1 and CLIC4 might complement CA125 as ovarian cancer tumor biomarkers. Figure 5c shows a high degree of complementarity of these biomarkers with CA125 as only one tumor (2% of all tumor cores) was negative for both CA125 and CLIC1 and only two tumors were negative for both CA125 and CLIC4.

CLIC1 and CLIC4 staining were not significantly affected by clinicopathological parameters such as age, race, disease stage, tumor grade, or tumor size (Table 1). In contrast, there was a significant difference in CA125 staining across tumor grade (p = 0.013) primarily because Grade 1 tumors were CA125 negative. However, these tumors were positive for both CLIC1 and CLIC4. Also, nearly 21% of early stage (stage I + II) EOC tumors were negative for CA125, whereas only 5% and 10% of these tumors were negative for CLIC1 and CLIC4, respectively. (Table 1). Finally, CA125, but not CLIC1 or CLIC4, showed a significant tumor subtype bias primarily because the mucinous tumors did not stain with CA125.

**Table 1:** Correlation of CLIC1, CLIC4 and CA125 with clinical characteristics in ovarian cancer patients.

Variables	CLIC1, n (%)		P-value*	CLIC4, n (%)		P-value*	CA125, n (%)		P-value*
Age	Negative	Positive		Negative	Positive		Negative	Positive	
<50	2 (25%)	6 (75%)	0.267	1 (12.5%)	7 (87.5%)	0.237	3 (37.5%)	5 (62.5%)	0.405
>=50	5 (10.6%)	42 (89.4%)		1 (2.1%)	46 (97.9%)		11 (23.4%)	36 (76.6%)	
Race									
Caucasian	4 (8.5%)	43 (91.5%)	0.055	2 (4.3%)	45 (95.7%)	1.000	12 (25.5%)	35 (74.5%)	1.000
Non-Caucasian	3 (37.5%)	5 (62.5%)		0 (0%)	8 (100%)		2 (25%)	6 (75%)	
Stage									
I + II	2 (10.5%)	17 (89.5%)	1.000	1 (5.3%)	18 (94.7%)	1.000	4 (21.1%)	15 (78.9%)	0.749
III + IV	5 (14.7%)	29 (85.3%)		1 (2.9%)	33 (97.1%)		9 (26.5%)	25 (73.5%)	
Grade									
1	0 (0%)	3 (100%)	1.000	0 (0%)	3 (100%)	1.000	3 (100%)	0 (0%)	0.013
2	1 (9.1%)	10 (90.9%)		0 (0%)	11 (100%)		1 (9%)	10 (91%)	
3	5 (17.2%)	24 (82.8%)		1 (3.4%)	28 (96.6%)		6 (20.7%)	23 (79.3%)	
Tumor size									
≥100	2 (11.1%)	16 (88.9%)	0.639	1 (5.6%)	17 (94.4%)	1.000	4 (22.2%)	14 (77.8%)	1.000
<100	3 (20%)	12 (80%)		0 (0%)	15 (100%)		3 (20%)	12 (80%)	
Subtype									
CLEAR	2 (40%)	3 (60%)	0.321	0 (0%)	5 (100%)	0.571	1 (20%)	4 (80%)	<0.0001
ENDO	0 (0%)	6 (100%)		0 (0%)	6 (100%)		0 (0%)	6 (100%)	
M/R	1 (8.3%)	11 (91.7%)		0 (0%)	12 (100%)		3 (25%)	9 (75%)	
MUCINOUS	0 (0%)	6 (100%)		1 (16.7%)	5 (83.3%)		6 (100%)	0 (0%)	
SEROUS	4 (15.4%)	22 (84.6%)		1 (3.8%)	25 (96.2%)		4 (15.4%)	22 (84.6%)	

*P-value was obtained from Fisher’s exact test.

As noted above, CLIC1 and CLIC4 were initially identified as blood biomarkers of EOC because human CLIC1 and CLIC4 were shed into the blood of xenografted tumor-bearing mice and significantly elevated in sera of EOC patients^25,27^. However, it was not clear from these earlier studies whether these proteins were produced and shed into the blood by all EOC subtypes as only one serous and one endometrial cell line were used in the initial discovery studies and most serum samples tested were from patients with high grade serous tumors. Because the above IHC analyses showed that CLIC1 and CLIC4 stain all EOC tumor subtypes, we next evaluated whether these proteins are also shed by all tumor subtypes. Fresh surgical specimens from patients representing the five major EOC subtypes were minced and placed in short term organ culture followed by in-depth LC-MS/MS quantitative comparisons of secretomes. As shown in Fig. 5d, all tested malignant tumors secreted CLIC1 and CLIC4 into the media and shedding was usually higher than from benign tumors. In contrast, CA125 shedding from benign and malignant tumors was far more variable, and a substantial number of malignant tumors showed negligible secretion of CA125 (Fig. 5e).

### Elevated CLIC4, but not CLIC1, is a negative indicator of patient survival

**Figure 6:**
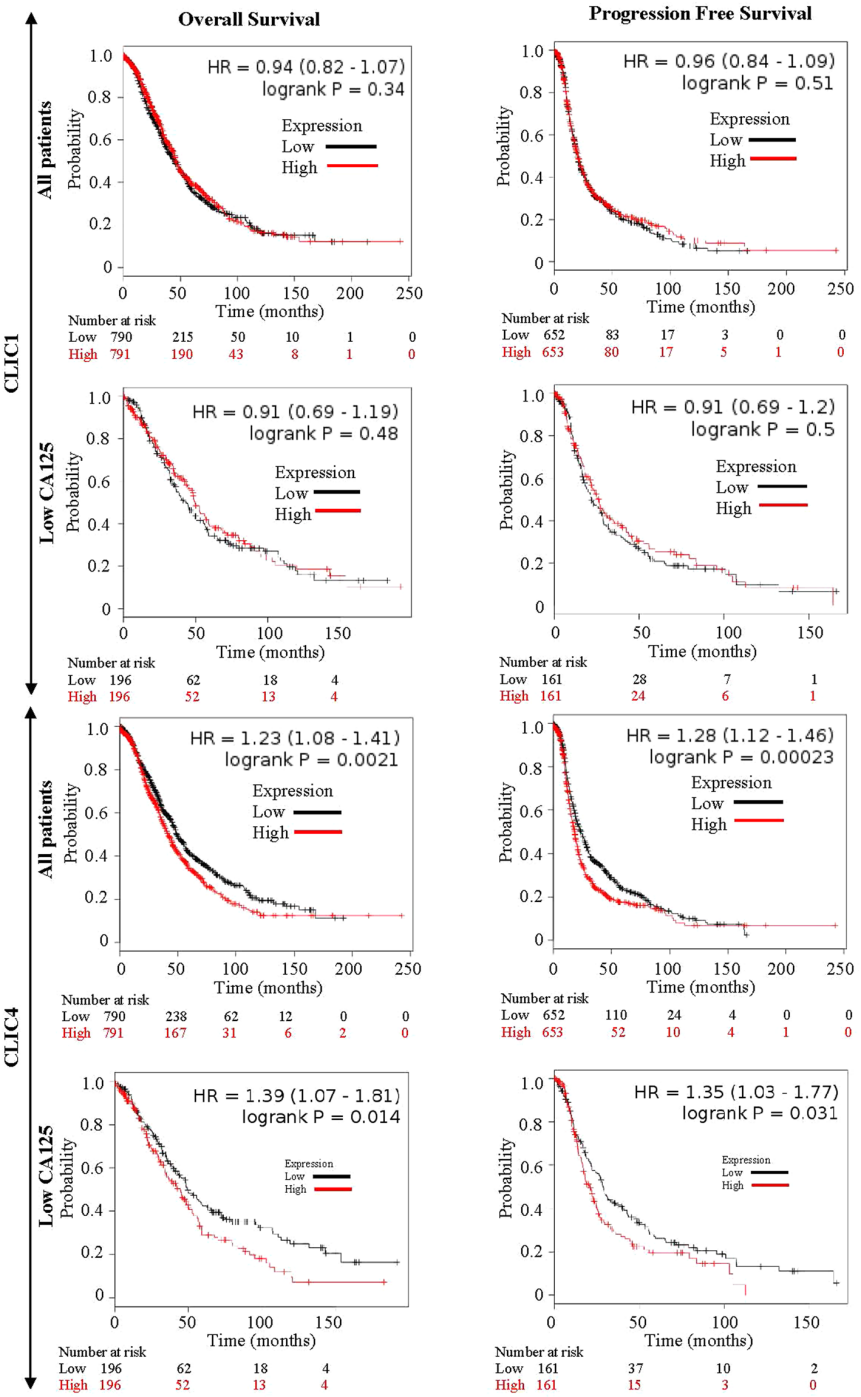
Elevated CLIC4, but not CLIC1, is a negative indicator of patient survival. Upper panels – CLIC1 mRNA levels correlated with overall and progression free survival in all EOC patients and patients with low CA125 (bottom quartile). Lower panels – CLIC4 mRNA levels correlated with overall and progression free survival in all EOC patients and patients with low CA125 (bottom quartile).

To determine whether expression of CLIC1 or CLIC4 has prognostic implications, we performed a Kaplan Meier survival plot analysis for CLICs expression using an online software tool (www.kmplot.com/ovar)^46^. Figure 6 shows that CLIC4, but not CLIC1, overexpression was associated with both decreased overall survival (p = 0.0021) and progression free survival (p = 0.00023) among EOC patients. Furthermore, among patients with low CA125 levels, elevated CLIC4 was also significantly negatively correlated with both overall survival (p = 0.014) and progression free survival (0.031).

### CLIC1 and CLIC4 are necessary for EOC cell proliferation and cell migration *in vitro*

**Figure 7:**
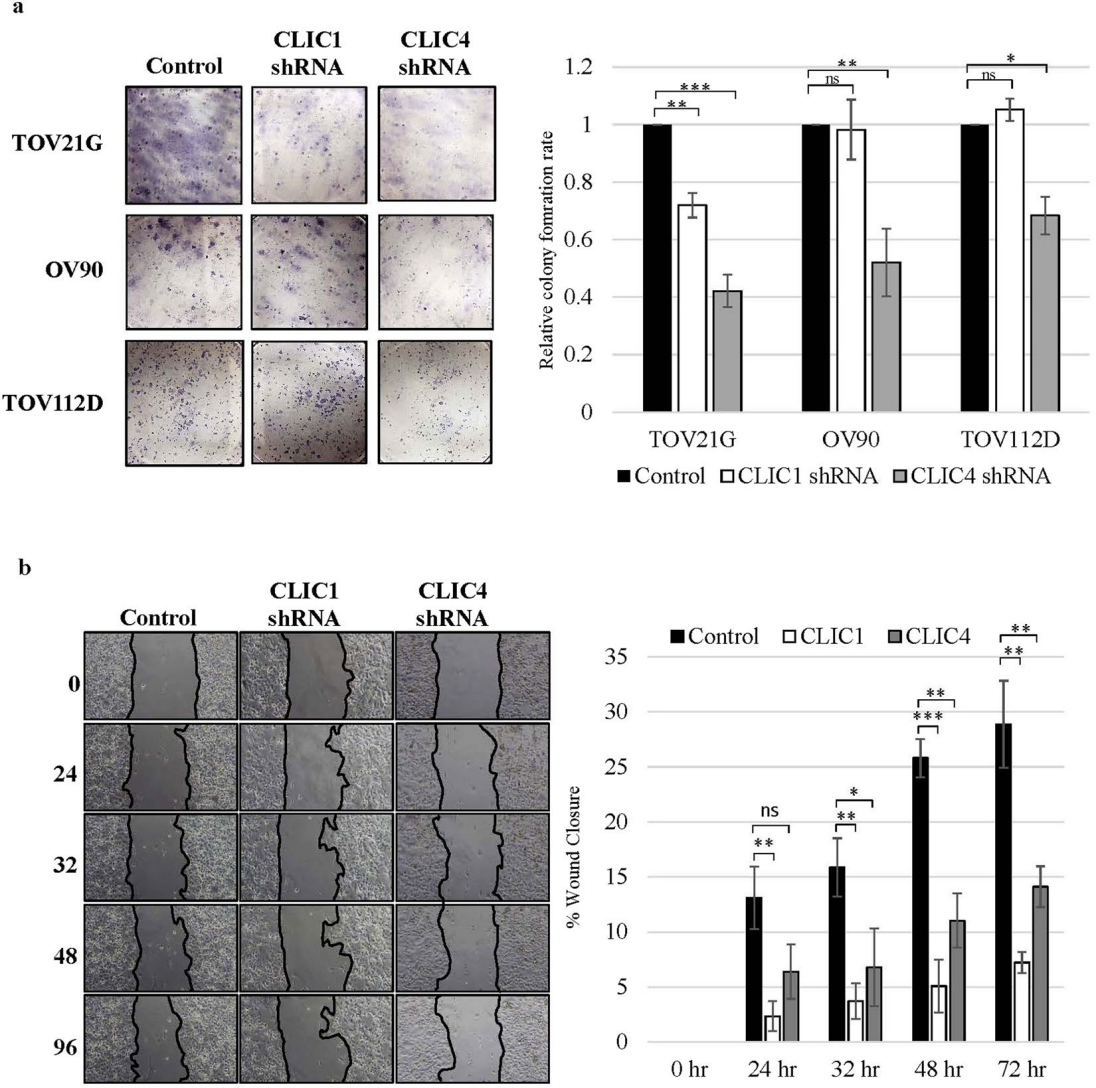
Knockdown of CLIC1 or CLIC4 decreases EOC cell proliferation and cell migration. TOV21G, OV90 and TOV112D cells were transduced with control, CLIC1 or CLIC4 shRNA. (**a**) Monoplast colony formation reflecting cell proliferation. Cells were stained with hematoxylin. (**b**) Wound healing assay to assess cell migration. Values represent the mean +/− SD of three independent experiments. Statistical significance was determined by performing ANOVA multiple comparisons, using GraphPad Prism 6 software (GraphPad Software, La Jolla, CA). ns = not significant. *p < 0.05; **p < 0.01; ***p < 0.001.

To assess the roles of CLIC1 and CLIC4 in EOC cell proliferation and migration, we evaluated the effects of separate knockdowns of these proteins using lentiviral shRNA in three EOC cell lines representing different subtypes (TOV21G – clear cell, OV90 – serous and TOV112D – endometrioid) (Supplemental Fig. 3). Knockdown of CLIC4 decreased cell proliferation and monoplast colony formation in all analyzed cell lines. In contrast, CLIC1 knockdown only decreased cell proliferation in TOV21G cells (Fig. 7a). Because the effects of knocking down CLIC1 and CLIC4 were most pronounced in TOV21G cells, we used this cell line to analyze migration in a wound healing assay. Knocking down either protein significantly decreased migration compared to control (Fig. 7b).

## Discussion

Epithelial ovarian cancer (EOC) is the most deadly gynecological cancer due in part to its asymptomatic nature at early stages and a lack of reliable minimally invasive diagnostic methods (for a recent review see ref.^47^). CA125 is currently used as a blood biomarker for monitoring EOC patients after diagnosis, but it lacks adequate sensitivity and specificity for early diagnosis or effective prognosis^9–14^. In addition, a number of biomarkers, such as WT1, p53, NAPSA, PGR, etc. are used at the tissue staining level to stratify diagnosed EOC cases into different subtypes^48,49^. While such previously known IHC biomarkers are quite valuable for determining EOC subtypes, our overall goal is to identify and validate biomarkers that improve diagnosis of EOC across all major subtypes, preferably at both the serum/plasma and tissue levels. In this study, we evaluated CLIC1 and CLIC4 staining of EOC in tissue microarrays as well as fresh tumor tissue secretomes to complement our prior human serum biomarker studies^25,27^. This is because there are two major barriers to validating new pan-EOC serum biomarkers. First, while proteomics can discover numerous new candidates, it is often impractical to validate new low abundance biomarkers in blood of large numbers of patients and controls unless robust ELISA assays are available. Second, serum and plasma sample sets from biorepositories typically are comprised primarily of high grade serous cases with very few specimens of the less common subtypes. IHC can provide additional insight as to whether proteins of interest are produced by all EOC subtypes and tumor secreteome analysis indicates whether these proteins are shed into the interstitial space where they are likely to be transferred to the blood.

In this study, a key finding is that at the tissue level either CLIC1 or CLIC4 stained larger percentages of the EOC tumor cores, across all major EOC subtypes, compared to CA125 (Fig. 5a,b and Table 1). Most notably, both CLIC1 and CLIC4 outperformed CA125 in early stage EOC tumors, grade 1 tumors and mucinous EOC subtype tumors (Figs 1–3 and 5b, Table 1), which are categories of tumors where CA125 performs particularly poorly as a serum biomarker. Although CLIC1 and CLIC4 are homologous proteins with 63% sequence identity, their staining patterns were quite distinct. CLIC1 staining of the malignant EOC tumors was observed only within the tumor nests but not in the adjacent stroma (Fig. 1), whereas CLIC4 stained both tumor nests and surrounding stroma (Figs 2 and 3). The presence of CLIC4 in the stroma was first described in breast cancer, where it appears to play a tumorigenic role through TGFβ-induced activation of tumor fibroblasts^50^. Likewise, in EOC, CLIC4 exhibits strong stroma and tumor cell staining and plays a possible role in TGFβ-mediated fibroblast activation^29^. Another study of multiple cancer types further confirmed the presence of CLIC4 in stroma surrounding the tumor nests. That study described decreased CLIC4 levels in the tumors and a subsequent increase in the stromal CLIC4 in the advanced tumors^51^. We observed a similar gradual reduction of nuclear CLIC4 staining and increased cytoplasmic staining with increased malignancy, suggesting tumor progression-related changes in intracellular CLIC4 localization (Fig. 3a,b). Taken together these results suggest that CLIC4 could serve as a marker for EOC tumor staging and possibly prognosis. Although CLIC4 also stained benign and normal ovarian tissue the staining pattern was markedly different as only light staining restricted to nuclei was observed. In contrast, as noted above, nuclear CLIC4 staining was only observed in some stage I tumors, whereas staining was mostly cytoplasmic in stage II, III and IV EOC tumors (Fig. 3b).

Consistent with broad staining across nearly all EOC tumor cores, CLIC1 and CLIC4 were also shed more consistently and at higher levels from fresh human tumors across tumor subtypes than CA125 (Fig. 5d,e). Because CLIC4 levels in serum from patients with benign tumors^27^ and in benign tumor secretomes (Fig. 5d) are typically lower than in patients with malignant tumors, we hypothesize that the intracellular location of CLIC4 might be an important factor in determining the extent to which the protein is shed into the interstitial space and subsequently into the blood. That is, only cytoplasmic CLIC4, but not nuclear CLIC4 may be shed by the cells. An interesting anomaly is that while all mucinous tumors in the tissue microarrays were negative for CA125 staining, a mucinous tumor secretome showed the highest level of CA125 shedding. Reevaluation of this specimen confirmed that it was classified correctly as the mucinous subtype by the pathologist.

As noted above, this study complements our prior pilot validation of CLIC1 and CLIC4 as promising serological biomarkers. We hypothesize that the most specific blood biomarkers are likely to be those proteins produced by the tumor and shed into the interstitial space and subsequently the blood at higher rates than normal or benign tissue. Of course, broad-based tissue staining and the presence of these proteins in tumor secretomes do not necessarily prove that they would be good serological biomarkers of EOC across subtypes. In addition to shedding by tumors, efficiency of transfer to the blood and half-life therein will affect observed serum/plasma biomarker levels. However, as noted above, our prior studies showed that CLIC1 and CLIC4 were shed by human tumor cells into the blood in xenograft mouse models, and these proteins were elevated in the blood of EOC patients compared with normal donors or patients with benign ovarian tumors^25–27^. While the prior studies indicated CLIC1 and CLIC4 should be good blood biomarkers of high grade serous EOC, the current data strongly suggest they are biomarkers of all EOC subtypes as well as early stage tumors (Table 1). This broad specificity is likely to extend to serum or plasma for all subtypes as the efficiency to transfer to the blood after shedding to the interstitial environment and blood half-lives are unlikely to vary greatly between subtypes. Therefore, the results reported here combined with the prior serological studies strongly suggest that CLIC1 and CLIC4 are promising tissue and serological biomarkers of all EOC subtypes, thereby justifying the substantial cost of developing high throughput serum/plasma assays. Development of these assays are currently being pursued.

Finally, the effects of CLIC1 and CLIC4 on tumor growth and migration (Fig. 7) suggest that they may be therapeutic targets and should be further investigated. As noted above, possible functional roles in tumor progression are incompletely understood and may differ depending upon the tumor type. In EOC, CLIC1 may be involved in cell cycle regulation and drug resistance^30^, while CLIC4 may have roles in fibroblast activation^29^. While CLIC1 has generally been associated with tumor progression in prior studies^32,34,38^, there are some contradictions regarding the role of CLIC4 in tumors. Our data suggest that inhibiting CLIC4 in EOC cell lines decreases cell proliferation and metastasis (Fig. 7), and that elevated tumor CLIC4 correlates with reduced patient survival (Fig. 6). Consistent with this are studies of head and neck cancers and human gliomas where CLIC4 silencing decreased cell proliferation and increased apoptosis^34,42^. Taken together, these results suggest that CLIC4 in cancer cells may support tumor growth and progression. However, CLIC4 appears to have an opposite role in lung^45^ and cutaneous squamous cell cancer^44^, which have low CLIC4 levels, and in these tumors increased expression of CLIC4 attenuated tumor growth and proliferation both *in vitro* and *in vivo*. The discrepancy about whether CLIC4 induces or inhibits tumor growth might be dependent upon the subcellular localization of CLIC4 in tumor cells, at least for some tumor types. In murine keratinocytes, it was observed that increasing the nuclear translocation of CLIC4 by mutating or deleting the N-terminus of CLIC4, increased apoptosis, whereas cytoplasmic accumulation lowered apoptosis^52^. Similarly, in cutaneous squamous cell cancer, nuclear localization of CLIC4 was associated with increased TGF-β-dependent transcriptional activity and tumor growth inhibition whereas the absence of nuclear CLIC4 enhanced tumor development^44^. Thus, it is possible that the role of CLIC4 in tumors depends both upon its distribution between stromal and tumor cells and its subcellular location, especially in the tumor cells. That is restriction of CLIC4 to the cytoplasm and not the nucleus of the tumor cells may increase cell proliferation and tumor progression. Consistent with this hypothesis, our IHC data showed that the advanced EOC tumors had more cytoplasmic CLIC4 staining than the early stage tumors (Fig. 5b).

As noted above, the biological effects of CLIC1 may not depend on cellular localization but may be EOC subtype specific. We observed that knocking down CLIC1 had the most profound effect on clear cell (TOV21G) cell proliferation but not on a serous (OV90) or an endometrioid (TOV112D) cell line (Fig. 7a). In apparent contrast to our results with OV90 cells, Qu *et al*.^29^ showed that knockdown of CLIC1 in A2780 cells decreased cell proliferation, and these cells were thought to be of the serous subtype. However, recent in-depth proteome analysis of numerous EOC cell lines showed the A2780 cell proteome most closely resembles clear cell EOC cell proteomes and was most likely misclassified^53^. Whereas a gene expression analysis suggested that A2780 is probably not even ovarian in origin^54^.

In summary, CLIC1 and CLIC4 are novel EOC biomarkers for both tissue staining and potentially as serological biomarkers where they are likely to complement CA125 to improve the sensitivity and specificity of EOC diagnosis. Combined analysis of these three proteins by IHC could detect all subtypes, stages and grades of EOC cases, and this may extend to similar roles as plasma/serum biomarkers because they are shed by all tumor subtypes into the interstitial fluid and are elevated in serum and plasma of EOC patients. These two CLIC isoforms show distinctly different staining patterns in EOC tumors and appear to have somewhat different effects on tumor progression and cell proliferation. In patient tumors, high levels of CLIC4 correlates with decreased patient survival whereas CLIC1 appears to not be a survival risk factor.

## Methods

### Tumor microarray (TMA)

Archived formalin fixed paraffin embedded (FFPE) tissue samples were used to prepare tissue microarrays at the Helen F. Graham Cancer Center (HFGCC) at Christiana Care, Newark, DE under a research protocol approved by the Christiana Care Institutional Review Board (IRB). The IRB waived the need for informed consent for these FFPE tissues since they were deidentified and coded pathological specimens in accordance with OHRP federal research regulation 45 CFR46.116(d) allowing the Waiver of Informed Consent. All research involving human subjects was performed in accordance with relevant OHRP regulations and guidelines. The histopathological classification and malignant grade of the tumors were confirmed by pathologists at the Center for Translational Cancer Research, HFGCC, according to the International Federation of Gynecology and Obstetrics (FIGO) standards. For preparation of the tissue microarray (TMA) slides, either 2 (array II) or 4 mm (array I) cores were transferred from archived paraffin-embedded tissue blocks onto recipient slides. The TMA slides contained a total of 55 ovarian cancer tissue, 5 normal ovary, 4 fallopian tube and 11 benign ovarian tumor specimens.

### Immunohistochemistry (IHC)

The paraffin embedded TMA slides were deparaffinized by washing in xylene followed by rehydration. Antigens were retrieved by treating the slides with citrate buffer (pH 6.0) and endogenous peroxidase activity was quenched by 3% H_2_O_2_. Subsequently the TMAs were incubated with 3% BSA to prevent nonspecific antibody binding. After blocking, the TMAs were incubated with monoclonal antibodies against CLIC1 (1:450, ab77214), CLIC4 (1:100, ab183043) and CA125 (1:250, ab110640) overnight at 4 °C, followed by further incubation with HRP labeled anti-rabbit, anti-mouse or anti-goat secondary antibody as appropriate. TMAs were then treated with di-aminobenzidine, counter stained with Mayer’s hematoxylin, dehydrated and mounted in crystal mount medium. The TMA slides were then subjected to blind, unbiased analyses by co-author RD. The tissue staining was scored as 0, 1, 2 or 3 based on their intensities. 0 was considered negative while 1, 2 and 3 were considered positive. Scoring was performed by an expert pathologist in a blinded manner (RD). Images of tissue cores were obtained using a Nikon DS-Fi2 camera and were analyzed using NIS Elements software (version 4.13).

### Tumor Secretome Analysis

Fresh patient-derived epithelial ovarian cancer tumors, benign tumors, normal ovary tissue and plasma were obtained from patients through the Helen F. Graham Tissue Procurement Center with written informed consent under a human subject protocol for Tissue Procurement research approved by the Christiana Care IRB. This was a uniquely different set of specimens from the ones used in the TMA. All research involving human subjects was performed in accordance with relevant OHRP regulations and guidelines. All specimens were de-identified and coded information containing age and tumor location, stage and subtype were provided. All tumor tissue samples were placed in RPMI cell culture media on ice and were stored overnight. Tissue was subsequently minced into approximately 1 × 1 mm slices and incubated in RPMI media for 4 h at 37 °C. After incubation, the tumor tissue was pelleted at 500 × g for 2 minutes at 4 °C. The RPMI containing the secreted tumor proteins was removed and filtered using a Millipore 0.22 µm centrifugal filtration unit. Protein concentration was determined using the Thermo Scientific Modified Lowry Protein Assay Kit. Secretome samples were aliquoted, snap frozen, and stored at −80 °C. For proteomics analysis an aliquot of each secretome was thawed and 10 µg was electrophoresed 0.5 cm into an Invitrogen 10% Bis-Tris gel using MES running buffer. Gels were stained using a Colloidal Blue Staining Kit (Invitrogen). In-gel trypsin digestion was performed as previously described^55^. Trypsin digested samples were analyzed using an Q Exactive Plus Orbitrap mass spectrometer (Thermo Scientific) interfaced with a Nano-Acquity UPLC system (Waters). For each digest 1 µg was separated using a RP-HPLC BEH C18 nanocapillary analytical column (Waters) with a 4-hour gradient. The mass spectrometer was set to scan 400–2000 m/z. The full MS scans were collected at 70,000 resolution. MS/MS scans were obtained on the Top20 most abundant ions and dynamic exclusion was set to 30 seconds. Peptide match was set to preferred, and unassigned and singly charged ions were rejected. Data was processed using MaxQuant_1.5.2.8 using default parameters. Data was searched using UP081315_Human9606 forward database and an in-house contaminants database containing trypsin, keratins, etc. LC-MS/MS data were searched using Match Between Runs enabled with a 0.7 min matching time window and a 10 min alignment time window. Peptide and protein false discovery rates were each set at 1.0%. Contaminants and proteins identified by a single peptide were filtered from the dataset. Protein intensities were normalized based on tumor volume from which the secretome was derived.

### Cell culture

The human epithelial ovarian cancer cell lines TOV21G, OV90 and TOV112D were obtained from American Type Culture Collection (ATCC, Manassas, VA). All the cells were maintained in a 37 °C incubator with a 5% CO_2_−95% air atmosphere in a 1:1 mixture of MCDB 105 media and Medium 199 (Sigma-Aldrich, St. Louis, MO) supplemented with 10% fetal calf serum.

### Transduction with lentiviral shRNA

CLIC1 (sc-60400-V), CLIC4 (sc-105213-V) and control (sc-108080) lentiviral shRNA particles were purchased from Santa Cruz Biotechnology (Santa Cruz, CA). Before transduction, 2.5 × 10^4^ cells were plated into a 24-well plate and incubated overnight. For each transduction, 5 × 10^4^ viral particles of either CLIC1, CLIC4 or control shRNA were used. For optimum transduction, 5 µg/ml polybrene was added to media containing 10% FCS. After 16–18 hours, the polybrene containing media was replaced with normal media containing FCS. The cells were allowed to grow for the next 48 hours. The non-transduced cells were selected using media containing 4 µg/ml puromycin.

### Protein extraction and Western Blot

Cells at approximately 80% confluence were harvested by washing *in situ* three times with Hanks Balanced Saline Solution followed by lysis in a Tris-SDS lysis buffer supplemented with protease inhibitors. The cell lysate was briefly sonicated on ice to shear DNA, centrifuged to sediment insoluble material and the protein concentration of the supernatant was determined using a BCA assay. For western blot, approximately 7 µg of the protein extract was separated on a 10% NuPage gel and transferred to PVDF membranes. The membranes were incubated with monoclonal antibodies against CLIC1 (ab77214) or CLIC4 (ab110640) followed by incubation with HRP labeled anti-mouse or anti-goat secondary antibody. Staining with anti-GAPDH was used as a loading control. Chemiluminescence was detected using a GeneGnome XRQ-NPC System (Synoptics, Fredrick, MD) and bands were quantitate using ImageJ (NIH) software.

### Monoplast colony formation assay

Approximately 1500 cells were plated for each experimental condition into 6-well plates. The cells were grown continuously in 2 ml media containing 10%FCS for 10 days. The cells were fixated with methanol and stained with hematoxylin for 30 minutes. Numbers of visible colonies were counted and the rate of colony formation was calculated by the formula: Colony formation rate = (number of colonies/number of cells plated) × 100.

### Wound healing assay

Approximately 500,000 cells were plated for each experimental condition into 6-well plates. The cells were allowed to form a monolayer using media with 10% FCS. After monolayer formation, the cells were serum starved for 24 hours and then scratched with a 200 µl pipette tip. The cells were washed, and fresh serum free media was added to each well. The wounds were monitored using an Eclipse Ts2 microscope with Nikon Imaging Source camera at 0, 24, 32, 48 and 72 hours. The area of the wounds for all time points were determined using ImageJ.

### Statistical Analysis

To analyze the correlation between the biomarkers’ expression and clinicopathological parameters Fisher’s exact test was performed using the StataCorp 13 (Stata Statistical Software, College Station, TX) software. For the *in vitro* assays, the results represent three independent experiments. To analyze the statistical significance of the *in vitro* data, ANOVA multiple comparisons was performed using GraphPad Prism 6 software (GraphPad Software, La Jolla, CA). P-values less than 0.05 were considered statistically significant for all the statistical analyses.

## Electronic supplementary material

Supplementary Information
