# Supplementary material for: CLIC1 and CLIC4 complement CA125 as a diagnostic biomarker panel for all subtypes of epithelial ovarian cancer
Source: Sci Rep. 2018 Oct 3;8:14725. doi: 10.1038/s41598-018-32885-2 (PMC6170428; doi:10.1038/s41598-018-32885-2)
Supplement: Supplementary file 1 — Supplementary Information [file 41598_2018_32885_MOESM1_ESM.pdf]

SUPPLEMENTARY INFORMATION

CLIC1 and CLIC4 complement CA125 as a diagnostic biomarker panel for all subtypes of epithelial ovarian cancer

Bipradeb Singha[1], Sandra Harper[1], Aaron R Goldman[1], Benjamin G. Bitler[2], Katherine M. Aird[3], Mark Borowsky[4], Mark Cadungog[4], Qin Liu[1], Rugang Zhang[5], Stephanie Jean[4], Ronny Drapkin[6], David W Speicher[1,*]

[1]Molecular and Cellular Oncogenesis Program, The Wistar Institute, Philadelphia, Pennsylvania, 19104, USA.[2]Department of Obstetrics and Gynecology, University of Colorado, Aurora, Colorado, 80045, USA. [3]Department of Cellular and Molecular Physiology, Penn State College of Medicine, Hershey, Pennsylvania, 17033, USA. [4]Helen F. Graham Cancer Center & Research Institute, Newark, Delaware, 19713, USA. [5]Gene Expression and Regulation Program, The Wistar Institute, Philadelphia, Pennsylvania, 19104, USA. [6]Department of Obstetrics and Gynecology, Ovarian Cancer Research Center, University of Pennsylvania Perelman School of Medicine, Philadelphia, Pennsylvania, 19104, USA.

Supplementary Figures

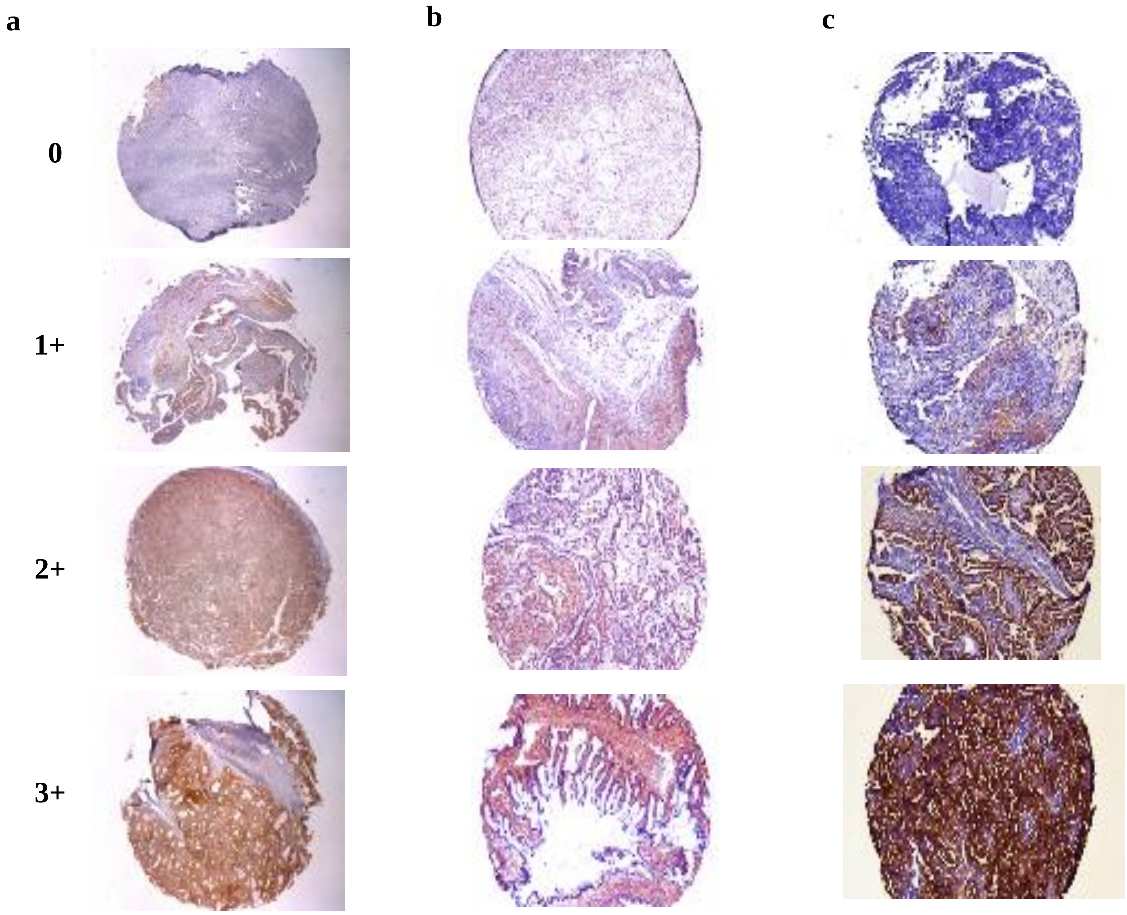

Figure S1: Representative images showing the different staining intensities for (a) CLIC1, (b) CLIC4 and (c) CA125 among the ovarian tumors. Staining intensity corresponding to 0 was considered negative for the study. 1+, 2+ and 3+ were considered positive.

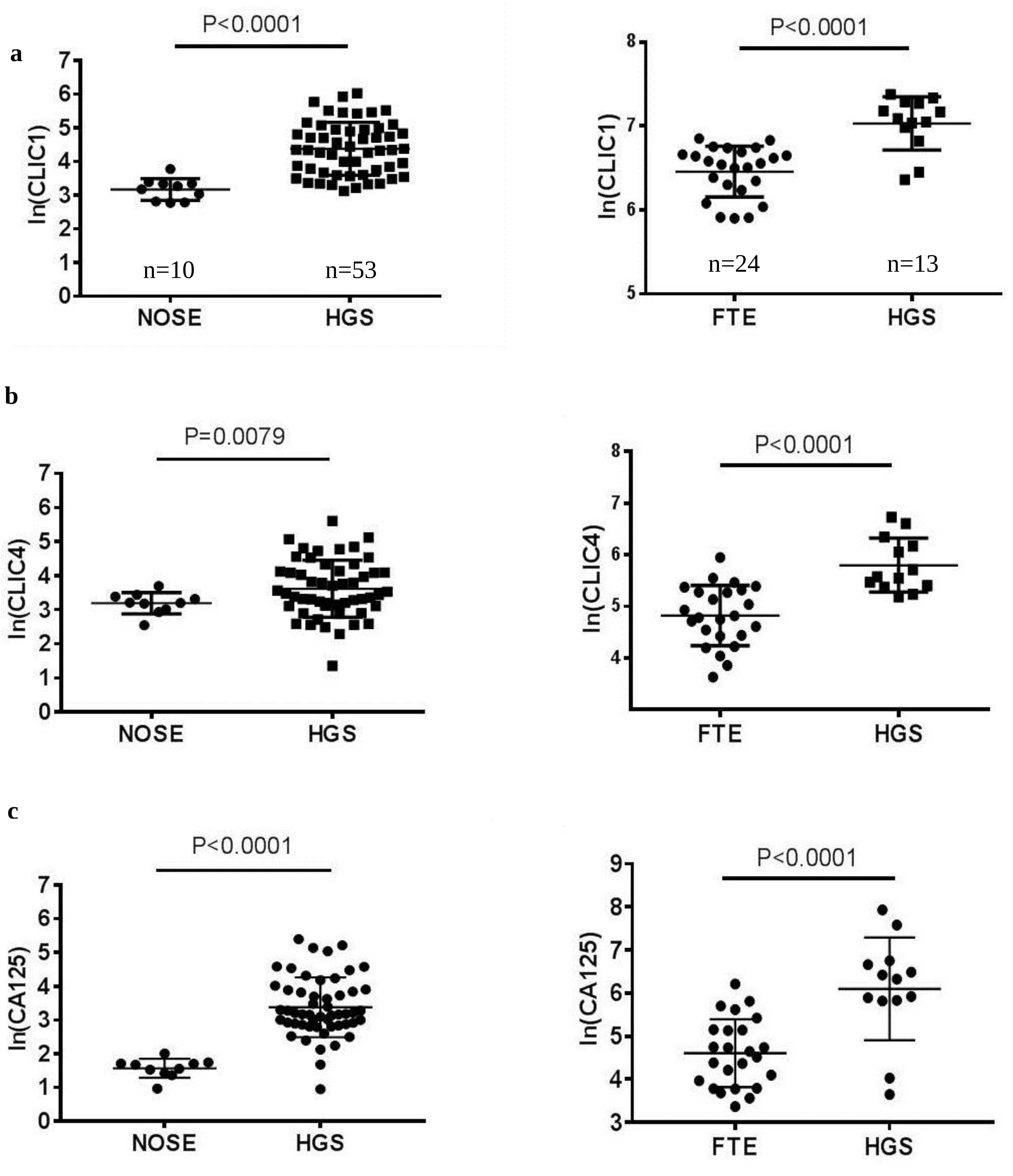

Figure S2: Meta-analysis of (a) CLIC1, (b) CLIC4 and (c) CA125 mRNA levels in GEO (gene expression omnibus) dataset GSE18520 (left) and GSE10971 (right). NOSE = normal ovarian surface epithelium, FTE = fallopian tube epithelium and HGS = high grade serous. p values were determined using unequal variance (Welch) t-test on log transformed mRNA levels of (a) CLIC1, (b) CLIC4 and (c) CA125.

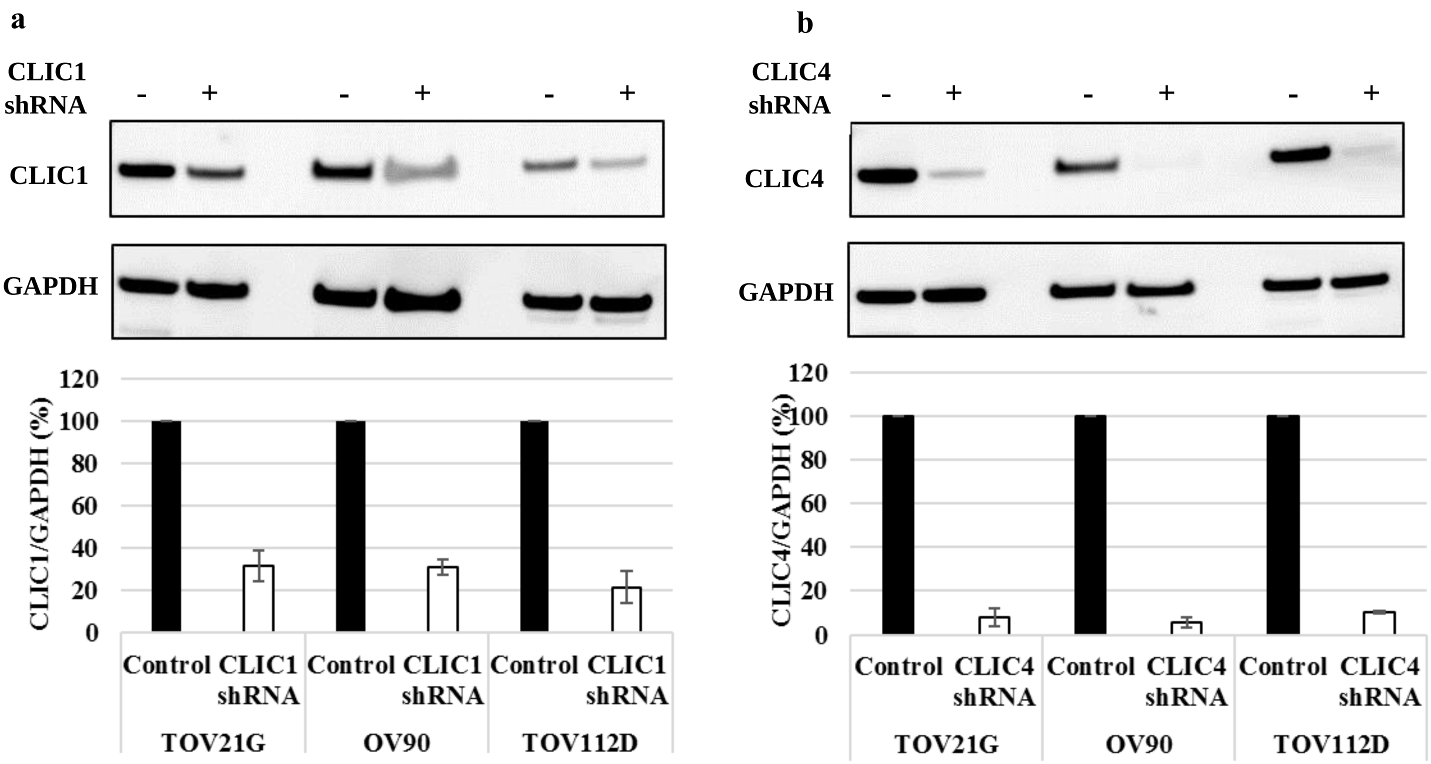

Figure S3: Knockdown of CLIC1 and CLIC4. Lentiviral shRNA targeted against CLIC1 or CLIC4 resulted in decreased levels of (a) CLIC1 and (b) CLIC4 respectively. After western blot analysis of the pertinent CLIC protein, the membrane was stripped and re-probed with antibody to GAPDH as a loading control. The corresponding full length western images are shown in Figure S4. Lower panels: densitometric analysis of the protein band intensities represent replicates of three experiments. Error bars indicate ± S.D. Statistical significance was determined by performing ANOVA multiple comparisons, using GraphPad Prism 6 software (GraphPad Software, La Jolla, CA). Asterisks denote statistically significant changes (***$p < 0.001$).

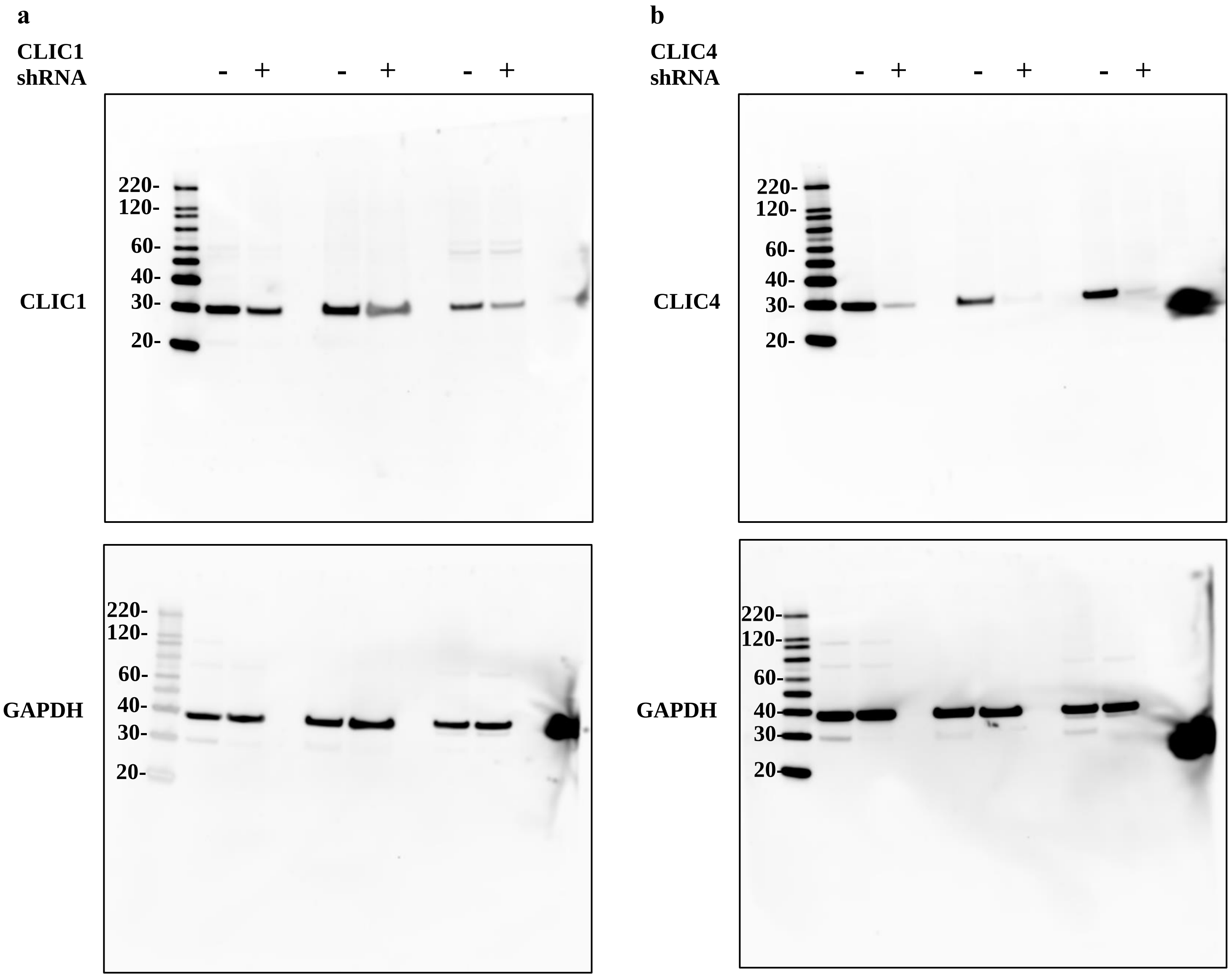

Figure S4. Full-length western blot images used for cropped images shown in Figure S3. Membranes were probed with respective CLIC antibodies, imaged, then stripped and re-probed using a GAPDH antibody. The left lane is a MagicMark protein standard mix.

Array-Position	Subtypes	Stage	Grade
I-A1	Benign	-	-
I-A2	Benign	-	-
I-A3	Mixed/Rare	2	3
I-A4	Endometrioid	1	ND
I-B1	Mixed/Rare	3	3
I-B2	Mixed/Rare	3	3
I-B3	Serous	3	3
I-B4	Serous	3	3
I-B5	Endometrioid	4	3
I-C1	ClearCell	4	ND
I-C2	Serous	2	3
I-C3	Mixed/Rare	2	3
I-C4	Serous	3	3
I-C5	Mixed/Rare	3	ND
I-D1	Serous	4	3
I-D2	Mixed/Rare	2	ND
I-D3	Serous	3	3
I-D4	Mixed/Rare	2	3
I-D5	Endometrioid	3	3

Array-Position	Subtypes	Stage	Grade
II-A1	Ovary	-	-
II-A2	FT	-	-
II-A3	Ovary	-	-
II-A4	FT	-	-
II-A5	Ovary	-	-
II-A6	FT	-	-
II-A7	Ovary	-	-
II-A8	FT	-	-
II-A9	Ovary	-	-
II-B1	Benign	-	-
II-B2	Benign	-	-
II-B3	Benign	-	-
II-B4	Benign	-	-
II-B5	Benign	-	-
II-B6	Benign	-	-
II-B7	Benign	-	-
II-B8	Benign	-	-
II-B9	Benign	-	-
II-C1	Mixed/Rare	3	3
II-C2	Mixed/Rare	3	2
II-C3	Endometrioid	1	ND
II-C5	Serous	4	3
II-C7	Serous	3	3
II-C8	Serous	3	3
II-C9	ClearCell	2	ND
II-C10	ClearCell	4	2
II-D1	Mucinous	3	3
II-D2	Serous	3	3
II-D3	Serous	1	2
II-D4	Mucinous	1	1
II-D5	Serous	4	2
II-D6	Serous	2	3
II-D7	Mucinous	1	ND
II-D8	Mixed/Rare	4	ND
II-D9	Mixed/Rare	3	ND
II-D10	Mucinous	ND	1
II-E1	Serous	3	2
II-E2	Serous	4	3
II-E3	Serous	4	ND
II-E4	Serous	3	3
II-E5	Serous	3	3
II-E6	Serous	ND	ND
II-E7	Serous	3	3
II-E8	ClearCell	1	3
II-E9	Serous	2	2
II-E10	Endometrioid	2	2
II-F1	Endometrioid	1	2
II-F2	Mucinous	1	1
II-F3	ClearCell	1	3
II-F4	Serous	3	3
II-F5	Mucinous	1	3
II-F6	Mixed/Rare	3	2
II-F7	Serous	4	ND
II-F8	Serous	3	2
II-F9	Serous	3	3
II-F10	Serous	3	2

Table S1: Details of the TMA arrays that were used for the study. The first TMA array, denoted here as I, contained 19 cores. Each core was 4 mm in diameter. The second TMA array indicated as II, contained 58 cores. The tissues in TMA-II were 2 mm in diameter. ND- not determined. FT- Normal fallopian tube.
